# The precise midline myelotomy through anatomical posterior median septum by dissecting dorsal column in microsurgical resection of ependymoma (2-dimensional operative video)

**DOI:** 10.3171/2023.7.FOCVID2317

**Published:** 2023-10-01

**Authors:** Jun-Hoe Kim, Chun Kee Chung

**Affiliations:** 1 Department of Neurosurgery, Seoul National University Hospital, Seoul;; 2 Department of Neurosurgery, Seoul National University College of Medicine, Seoul; and; 3 Department of Brain and Cognitive Sciences, Seoul National University College of Natural Science, Seoul, Republic of Korea

**Keywords:** ependymoma, midline myelotomy, dorsal column, intramedullary spinal cord neoplasms, microsurgery

## Abstract

Although resection is the gold standard treatment for spinal ependymoma, permanent neurological deterioration has been reported postoperatively in 20%–27% of patients. Despite thorough dissection of the tumor from its surroundings, conventional longitudinally directed midline myelotomy can lead to injury to the dorsal column, possibly due to deformation of the posterior median septum as the tumor grows. To address this issue, the authors have been performing precise midline myelotomy through the anatomical posterior median septum by directly dissecting the dorsal column. This video presents the principles and application of this technique.

## Transcript

This video is demonstration of a precise midline myelotomy through anatomical posterior median septum by dissecting dorsal column in microsurgical resection of ependymoma.

### 0:32 Clinical Presentation.

The patient was a 54-year-old female with a history of posterior neck pain for 2 weeks. Her neurological examination was notable for both hip flexion weakness. The examination also showed left forearm and hand hypesthesia. She was unstable when doing tandem gait and positive in Romberg test. MR image showed a large enhancing T2 hyperintense intramedullary mass at C3–5 level. Peritumoral edema was also observed. The lesion had clear demarcation and was located in the center of the spinal cord. These findings were mostly consistent with ependymoma.

### 1:20 Rationale for the Procedure.

We decided to resect the tumor surgically by posterior approach with midline myelotomy. Because resection is gold standard for treatment of ependymoma^1–4^ and posterior approach with midline myelotomy is safest and shortest corridor to approach centrally located intramedullary lesion. Especially we choose to dissect dorsal column directly when doing midline myelotomy because posterior median septum is not linear and not vertical. Further explanations of this will be covered on the next page.

### 1:59 Explanation on Deformation of Posterior Median Septum.

As you can see in the picture, the exposed posterior median septum is not linear. And the figure shows that the posterior median septum is oblique rather than vertical. Even in cases where the tumor is located centrally within the spinal cord and spinal cord rotation is not present, it is likely that the growth of the tumor caused deformation of the posterior median septum, resulting in an oblique orientation.

### 2:29 Risk of Procedure and Its Potential Benefit.

During midline myelotomy, there is a risk of dorsal column injury, and other additional neural damage may occur during dissection of the tumor. By dissecting the dorsal column during midline myelotomy, we can safely approach the lesion with minimal neural tissue damage, as mentioned earlier. Also, gross-total resection of spinal cord ependymoma could promote better progression-free survival and overall survival.^1–4^

### 3:03 Alternatives and Why They Were Not Chosen.

Observation might be a viable option due to the indolent natural course of ependymoma. However, considering that preoperative neurological status is related to postoperative functional outcomes, delaying surgery may not be the best option.^5^ And the role of radiotherapy or systemic therapy is limited.^1–4^

### 3:32 Description for the Setup (Positioning, Equipment).

The patient was positioned prone with head flexion. Intraoperative neuromonitoring, including SSEP, MEP, free-running EMG, was utilized throughout the operation.

### 3:47 Description for the Setup (Key Surgical Steps).

After midline skin incision and subperiosteal muscle dissection, laminotomy was performed with a high-speed drill. After the dura was exposed, midline durotomy and arachnoidotomy was performed. The rest of the procedures are going to be demonstrated in the operative video.

### 4:09 Midline Myelotomy (Dorsal Column Dissection).

To tell you about the process of midline myelotomy, first of all, we accurately delineated the posterior median sulcus by careful inspection of surrounding structures such as posterior median spinal vein, contour of dorsal column, and dorsal root entry zones at both sides. After pial membrane-only incision, precise dorsal column dissection was done, maintaining the structural integrity of the dorsal columns. Finally, the tumor was exposed after carefully dissecting the overlying dorsal column from the tumor.

### 4:46 Exposure of Spinal Cord.

After midline durotomy and arachnoidotomy, spinal cord was exposed.

### 4:51 Identification of Posterior Median Sulcus.

After coagulating the posterior median vein, pial membrane incision was done in midline and posterior median sulcus between bilateral dorsal columns was identified.

### 5:08 Identification of Posterior Median Septum.

As we dissected the dorsal columns from each other, posterior median septum became more prominent.

### 5:20 Dorsal Column Dissection.

Extending the posterior median septum developed previously, the dorsal column dissection for the planned level was done.

### 5:48 Exposure of Tumor.

After that, the tumor was shown behind the ependymal lining.

### 5:55 Deformation of Posterior Median Septum.

The posterior median septum was deviated to the right because tumor was sprouted from left side, so the dorsal surface of tumor was covered by left dorsal column.

### 6:09 Dissection of Overlying Left Dorsal Column.

When you dissect the overlying dorsal column, particular attention should be paid for dorsal column injury.

### 6:24 Identification of Caudal End of Tumor.

After the entire length of the tumor was exposed, the caudal end of the tumor was identified.

### 6:36 En Bloc Removal of the Tumor.

From caudal to cephalad, the tumor was dissected from surrounding tissue and en bloc removal of the tumor was done.

### 6:53 Preserved Bilateral Dorsal Column.

After tumor removal, bilateral dorsal column was intact. Intraoperative neuromonitoring such as MEP and SSEP show no change until the very end of surgery.

### 7:08 Closure.

After meticulous hemostasis, pial edges and arachnoid membrane were reapproximated, using several interrupted 8-0 Prolene sutures. Primary dura closure was performed with 6-0 Prolene running sutures.

### 7:33 Disease Background: Ependymoma.

Ependymoma is the most common intramedullary glial tumor in the spinal cord of adults.^6–8^ WHO grade II or III comprise up to 75% and 11% of all adult spinal ependymoma cases, respectively.^9^ Surgery is the gold standard of treatment for spinal ependymoma because gross-total resection is related to better prognosis, and the role of adjuvant radiotherapy and systemic therapy is limited.^1–4^ Although gross-total resection is a primary target of surgery, permanent neurological decline was observed 20%–27% of patients.^5,6^

### 8:18 A Brief Review of Outcome.

After the surgery, the patient maintained good swallowing and gait functions. Bilateral hip flexion motor power was improved from grade 4 to grade 5. There is nearly no newly developed neurological deficit postoperatively except for bilateral foot hypesthesia. Postoperative contrast-enhanced MR image revealed total resection of the tumor without any complicating features.

## Disclosures

The authors report no conflict of interest concerning the materials or methods used in this study or the findings specified in this publication.

## Author Contributions

Primary surgeon: Chung. Assistant surgeon: Kim. Editing and drafting the video and abstract: both authors. Critically revising the work: both authors. Reviewed submitted version of the work: both authors. Approved the final version of the work on behalf of both authors: Chung. Supervision: Chung.

## Supplemental Information

### Patient Informed Consent

The necessary patient informed consent was obtained in this study.
